# Beyond the apnea-hypopnea index: a call to action for a multidimensional evaluation in obstructive sleep apnea

**DOI:** 10.3389/frsle.2026.1881431

**Published:** 2026-08-31

**Authors:** Peter C. Gay, Luigi Taranto-Montemurro, Lenise J. Kim, Ali Azarbarzin

**Affiliations:** 1 Division of Pulmonary, Critical Care, Sleep and Allergy Medicine, Mayo Clinic, Rochester, MN, United States; 2 Apnimed, Inc., Cambridge, MA, United States; 3 Department of Anesthesiology and Critical Care Medicine, The George Washington University, Washington, DC, United States; 4 Division of Sleep and Circadian Disorders, Brigham and Women's Hospital and Harvard Medical School, Boston, MA, United States

**Keywords:** chronic intermittent hypoxia, clinically meaningful, CPAP, hypoxic burden, obstructive sleep apnea

## Abstract

Obstructive sleep apnea (OSA) is a highly heterogeneous chronic disease, in which the conventional measure of disease severity, the apnea–hypopnea index (AHI), correlates poorly with patient-reported symptoms and inconsistently with cardiovascular outcomes. The present perspective proposes an initial discussion of a new concept for multidimensional evaluation of patients with OSA that integrates both objective measures of upper airway obstruction (AHI) and chronic intermittent hypoxia (hypoxic burden), along with subjective assessments of nighttime and daytime symptoms burden including snoring, subjective sleep quality, daytime sleepiness, fatigue, and quality of life. This integrated approach aims to improve characterization of patients and guide more individualized therapeutic goals, including improvements in quality of life and reduction in cardiovascular and metabolic consequences. It also supports a call to action in the OSA field, encouraging the development of a more comprehensive assessment of treatment response and clinical meaningfulness in OSA.

## 1 Obstructive sleep apnea: a heterogeneous disease defined by a single metric

Obstructive sleep apnea (OSA) is a chronic disease affecting nearly 1 billion people worldwide and >80 million adults in the United States (Benjafield et al., 2019; Sönmez et al., 2025). OSA is a major health problem linked to cardiometabolic comorbidities and increased risk of mortality (Young et al., 2008; Punjabi et al., 2009; Javaheri et al., 2024; Punjabi et al., 2002). OSA is a heterogenous disease, in which a substantial variability in physiological traits, symptom burden, cardiovascular risk, and treatment responses are observed. OSA affects all ages, sex, and levels of body mass index (Peppard et al., 2013; Esmaeili et al., 2025; Cunningham et al., 2021; Gray et al., 2017). The pathophysiology of OSA is multifactorial, involving anatomical predispositions, upper airway neuromuscular dysfunction, ventilatory control instability, and low arousal threshold (Eckert, 2018; Eckert et al., 2013; Wellman et al., 2013, 2011). The presence and severity of symptoms do not consistently correlate with objective OSA severity and 30%−60% of patients with OSA are in fact asymptomatic or minimally symptomatic (Keenan et al., 2018; Ye et al., 2014), which further contributes to the complexity of OSA clinical presentation.

Despite its clinical heterogeneity, OSA has historically been defined and graded using a single metric, the apnea-hypopnea index (AHI), which quantifies the frequency of apneas and hypopneas per hour of sleep. AHI has been the cornerstone of diagnosis and classification of OSA severity. However, several limitations of AHI need to be considered. AHI as a frequency metric does not account for the contributions of different pathophysiological traits of OSA. AHI correlates only weakly with important clinical outcomes, such as daytime sleepiness, quality of life (QoL), and cardiovascular risk (Gottlieb et al., 1999; Redline et al., 2010; Shahar et al., 2001; Quan et al., 2014). AHI alone does not account for many pathophysiological dimensions of OSA severity, including the duration and depth of oxygen desaturation or degree of sleep fragmentation. Similarly, traditional single-metric definition of success (AHI < 5 events/h or a ≥50% AHI reduction) may not fully reflect meaningful improvements in pathophysiology or patient-centered outcomes (Azarbarzin et al., 2020; Pevernagie et al., 2020; Malhotra et al., 2021). Current payer coverage is dependent on AHI alone which may limit access to treatments in patients with different presentations of OSA.

In this sense, AHI is insufficient as a standalone metric to assess OSA severity and disease burden. Additional objective measures of respiratory disturbance and a comprehensive assessment of patient symptoms may better capture disease severity and inform treatment management. The Agency for Healthcare Research and Quality within the United States Department of Health and Human Services published a highly critical report of the Sleep Health Community in 2020. The document was a clear call upon sleep researchers to move beyond the AHI as a single descriptor of disease and to better justify continuous positive airway pressure (CPAP) treatment in particular as a therapy for clinically meaningful outcomes (Balk et al., n.d). Recent efforts, such as the Baveno classification, aim to move beyond AHI in OSA by evaluating both symptoms and comorbidities to better characterize disease burden (Randerath et al., 2021; Matthes et al., 2024). The Baveno system grades OSA based on a combination of symptom intensity and the presence of clinically significant comorbidities, such as resistant or uncontrolled hypertension, atrial fibrillation, heart failure, stroke, and diabetes. Patients are assigned to one of four groups (A–D): group A (minor symptoms and minor comorbidities), group B (severe symptoms and minor comorbidities), group C (minor symptoms and severe comorbidities), and group D (severe symptoms and severe comorbidities). These groups appear to differ in hypoxic load and patient-reported outcomes, and treatment benefit is focused on symptomatic and/or comorbid groups (Randerath et al., 2021). More recently, the modified Baveno classification has reincorporated a high-AHI threshold (≥30 events/h) and formal cardiovascular risk estimation to refine treatment indication (Matthes et al., 2024). While this new system requires further validation in longitudinal studies, it underscores limitations in current OSA assessment methods and the urgent need for clinical change.

**Figure 1:**
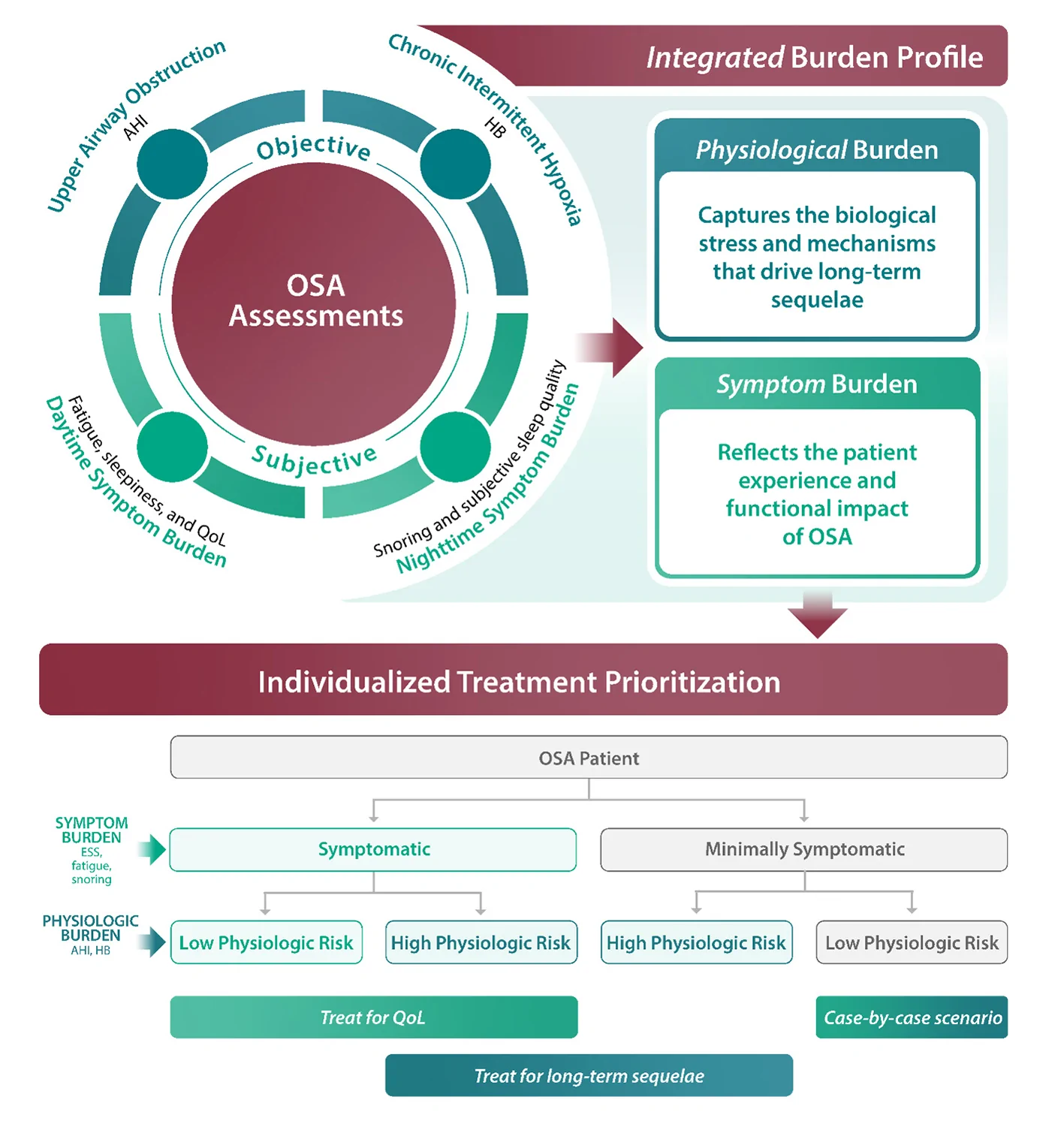
Multiple components of clinical meaningfulness in a proposed concept of multidimensional evaluation for a treatment decision matrix for obstructive sleep apnea (OSA). AHI, apnea hypopnea index; HB, hypoxic burden; QoL, quality of life; ESS: Epworth Sleepiness Scale.

Here, we issue a call to action to the field of sleep medicine for a multidimensional evaluation of OSA that integrates *objective* measures of pathophysiological burden, including upper airway obstruction and chronic intermittent hypoxia (CIH), and *subjective* patient-reported nighttime (e.g., snoring and subjective sleep quality) and daytime (e.g., daytime sleepiness, fatigue, and symptomatic burden) with the aim to improve OSA risk stratification and clarify the clinical meaningfulness of treatment effects (Figure 1). The objective and subjective components of this approach to OSA evaluation are further detailed in the sections that follow.

## 2 A concept of multidimensional evaluation OSA and treatment response

### 2.1 Objective measures of pathophysiologic burden

#### 2.1.1 Upper airway obstruction: AHI as a starting point, not the single endpoint

Observational studies have demonstrated associations between higher AHI and increased risk of cardiometabolic sequelae, including hypertension (Peppard et al., 2000), heart failure (Gottlieb et al., 2010), and stroke (Redline et al., 2010). However, there is conflicting evidence on how much a reduction in AHI values translates into decreased long-term risk of adverse clinical outcomes. Large, randomized controlled clinical trials have not consistently shown a reduction in cardiovascular events among patients using CPAP, the first-line treatment choice for OSA, with positive outcomes only shown in *post-hoc* analyses including more adherent patients (McEvoy et al., 2016).

Moderate and severe OSA, defined as AHI ≥ 15 events/h, are generally treated even in the absence of symptoms due to the observed association with adverse cardiovascular outcomes (Veasey and Rosen, 2019). In contrast, mild OSA (AHI < 15 events/h) is often untreated unless patients are symptomatic or have relevant cardiometabolic comorbidities (Chowdhuri et al., 2016). Of note, patients with the same “mild” AHI may differ markedly in the clinical meaningfulness of their respiratory events: 10 long apnea events/hour are not equivalent to 10 short hypopnea events/hour, despite both being classified as mild OSA (AHI = 10 events/h). The relationship between AHI and clinical outcomes is affected by the heterogeneity of respiratory events during different sleep stages. It has been shown that obstructive events occurring predominantly during rapid eye movement (REM) sleep are independently associated with higher incidence of cardiovascular events, insulin resistance, and type-2 diabetes despite patients with REM-related OSA may have relatively low AHI (Aurora et al., 2018; Bonsignore et al., 2024). Similarly, hypertension appears to be more strongly associated with OSA during REM sleep (Mokhlesi et al., 2014). As such, limited nightly CPAP use in the early portion of sleep may reduce measured AHI, but still fail to prevent REM-related disease burden and its downstream consequences. Incorporating REM-related information into a multidimensional evaluation may face some practical limitations. Electroencephalographic recordings may not be available for some modalities of home sleep testing, the first-line diagnostic approach in many healthcare systems, which could limit the assessment of REM-related metrics. However, recent technological advancements are making it possible to identify REM sleep based on photoplethysmography or respiratory signals collected with home sleep apnea tests (Korkalainen et al., 2020; Bakker et al., 2021). Traditionally, treatment success has been defined by reductions in AHI, ranging between normalization of AHI to < 5 events/hour for CPAP therapy or at least a ≥50% reduction from baseline for alternative therapies. This concept reflects the broader notion of disease control, defined as the extent to which treatment reduces OSA-related pathophysiologic disturbances and long-term clinical sequelae.

In clinical practice, achieving such targets leads to escalation of CPAP pressures to eliminate residual respiratory events. However, higher therapeutic pressures may negatively impact sleep quality and patient tolerance/adherence, potentially leading to treatment-related sleep disruption. Evidence has also indicated that exposure to high CPAP pressures may be associated with pulmonary inflammation and injury, raising concerns of potential downstream increased cardiovascular risk (Gottlieb et al., 2022; Peker et al., 2024). Importantly, the effectiveness of CPAP therapy is limited by poor adherence. Clinical practice data suggest that up to 61% of patients discontinue CPAP within the first year of treatment (Chai-Coetzer et al., 2013; Weaver and Grunstein, 2008). Even among patients who remain on therapy, adherence is often sub-optimal, with many patients using CPAP for ≤ 4 h/night (Rotenberg et al., 2016). Consequently, improvements in AHI measures during CPAP use may not fully reflect the residual burden of untreated disease through the entire sleep period.

Furthermore, reductions in AHI do not necessarily capture other dimensions of OSA pathophysiology. Residual hypoxemia and persistent symptoms may remain despite improvement in AHI (Tankéré and Taillard, 2025; Parekh, 2024). Conversely, a clinically meaningful therapeutic effect does not necessarily require complete normalization of AHI. Observational data suggest a progressive relationship between OSA severity and cardiovascular risk, hence further reductions in severity are associated with further lowering of cardiovascular risk (Gottlieb et al., 2010; Craciun et al., 2025). Accordingly, for patients on alternative therapies, success is defined when they achieve a ≥50% reduction in AHI, improvement in disease severity, or symptom relief. Alternative therapies, such as oral appliances, upper-airway surgery, hypoglossal nerve stimulation, and weight reduction provide clinical benefits to selected subgroups of patients (Park and Choi, 2025) and the effects on long-term cardiovascular risk remain unclear. These gaps reinforce the importance of assessing treatment response across multiple objective and symptomatic domains rather than by AHI reduction alone, regardless of the therapeutical intervention.

Despite its limitations, AHI remains the most widely used metric of OSA severity, providing a common language across clinical practice and decision-making. Thus, a more comprehensive assessment of OSA risk stratification and treatment response should consider the combination of AHI as an initial metric of upper airway obstruction in combination with the effects of therapy on symptom improvement, hypoxic exposure and resultant cardiovascular risk.

#### 2.1.2 CIH: hypoxic burden as a physiologic metric for risk

Weighing respiratory events by the depth and duration of oxygen desaturation provides a different physiologically meaningful assessment of OSA severity, compared to event count alone (i.e., AHI). Longer and deeper desaturations are associated with greater cumulative hypoxemia, higher CO_2_ retention, and larger intrathoracic pressure swings (Malhotra and White, 2002; Sands et al., 2018), thereby more clearly reflecting biological stress. Reoxygenation kinetics may further modulate downstream consequences, as delayed or incomplete recovery from desaturation can amplify oxidative stress. The severity and impact of hypoxemia may also vary across sleep stages. Greater oxygen desaturation is typically observed during REM-related OSA and has been associated with increased cardiometabolic risk (Mokhlesi et al., 2014). Given that patients with similar AHI may exhibit different hypoxic profiles and clinical outcomes (Kulkas et al., 2013), incorporation of oxygenation-based metrics may provide a more comprehensive assessment of disease burden and clinical meaningfulness.

Hypoxic burden (HB) has emerged as a robust marker of CIH. In contrast to metrics based on time and frequency [e.g., time spent below 90% saturation (T90) and oxygen desaturation index (ODI)], the HB integrates the frequency, depth, and duration of desaturations as the area under the desaturation curve (Azarbarzin et al., 2019). This integrative formulation allows area-based metrics to discriminate among patients with different nocturnal hypoxia profiles. Nevertheless, HB is not yet widely available in routine clinical practice, and other oxygenation metrics, such as ODI and T90, are alternatives for examining CIH, although their correlation with cardiovascular risk is less clear. The HB has been shown to predict cardiovascular outcomes and all-cause mortality more strongly than AHI (Azarbarzin et al., 2019; Trzepizur et al., 2022). As such, patients in the highest HB categories have an increased risk of cardiovascular-related mortality (Azarbarzin et al., 2019; Trzepizur et al., 2022; Peker et al., 2025), supporting the concept that cumulative hypoxic exposure is a key driver of adverse outcomes. In addition, HB appears to strongly predict symptoms related to OSA. Higher HB was independently associated with increased odds of excessive daytime sleepiness defined by an Epworth Sleepiness Scale (ESS) >10. Multiple studies have demonstrated that subjective sleepiness assessed by the ESS poorly correlates with AHI (Gottlieb et al., 1999; Lipford et al., 2019; Weaver et al., 2004), highlighting the symptom–severity dissociation with this OSA metric. Therefore, these findings may support HB as a better symptom-relevant marker of OSA.

HB may identify patients who are most likely to benefit from OSA therapy (Pinilla et al., 2023). A multi-trial analysis has shown that CPAP reduced cardiovascular risk primarily in patients with high-risk OSA (high baseline HB or heart rate response), with little or even adverse benefit in lower-risk OSA patients (Azarbarzin et al., 2025). These findings suggest that treatment effects may vary across OSA phenotypes, and in some patients, CPAP without substantial HB may offer no cardiovascular benefit and potentially introduce competing adverse physiological effects (e.g., pressure-related pulmonary stress), warranting further investigation. We propose that HB could be a key objective measure to integrate the evaluation of treatment response in OSA (Figure 1), assisting in the stratification of cardiovascular/mortality risk in patients with OSA, as well as in the assessment of improvement in patient-reported outcomes.

### 2.2 Subjective measures of patient-reported symptomatic burden

Patients with OSA can experience a broad range of symptoms during both nighttime (e.g., subjective sleep quality and snoring) and daytime (e.g., daytime sleepiness and fatigue,), (Gottlieb and Punjabi, 2020; Stepnowsky et al., 2019), although many OSA patients can remain asymptomatic (Keenan et al., 2018; Ye et al., 2014). The nighttime and daytime symptoms burden is further discussed in the sections that follow.

#### 2.2.1 Nighttime symptoms: snoring and subjective sleep quality

Snoring is one of the most common and often earliest nighttime manifestation of OSA as it is frequently the complaint that prompts patients or their bed partners to seek clinical evaluation. Snoring may carry independent clinical significance. Heavy or habitual snoring has been associated with carotid atherosclerosis independent of apnea severity (Lee et al., 2008). In a meta-analysis, habitual snoring was associated with modestly increased risks of ischemic stroke and coronary heart disease (Li et al., 2014). However, associations with overall cardiovascular disease and all-cause mortality remain unclear (Li et al., 2014; Rosen et al., 2019; Marshall et al., 2012), which in part could be related to the lack of a standardized approach to assessing snoring. Snoring also imposes a considerable psychosocial burden on the patients and their bed partner. Snoring is associated with marital problems and, in some studies, with higher levels of depressive and anxiety symptoms independently of OSA severity, sleep duration, and daytime sleepiness (Jeong et al., 2021; Luyster, 2017).

Sleep fragmentation is an important contributor to symptoms and impaired daytime functioning in OSA; however, arousal related indices have shown limited incremental value over AHI for cardiovascular risk prediction in most analyses (Trzepizur et al., 2022; Marin et al., 2005; Azarbarzin et al., 2023). Key limitations are that standard scoring defines arousals as binary EEG events, despite wide variation in magnitude and physiologic impact (American Academy of Sleep Medicine, 2023; Berry et al., 2017), and clinical scoring is subject to substantial inter-scorer variability (Rosenberg and Van Hout, 2013). These considerations support moving beyond simple arousal counts toward patient-reported sleep quality metrics, but this issue is complex and remains ill-defined. Clinically, such metrics may be most useful for identifying patients in whom fragmented sleep and insomnia-like complaints are prominent, including the comorbid insomnia and sleep apnea (COMISA) phenotype and those with subclinical insomnia. Insomnia symptoms are reported by roughly 22%−36% of patients with OSA, depending on the cohorts analyzed and the different definitions of OSA or insomnia (Zhang et al., 2019; Sweetman et al., 2021b). COMISA has been associated with a higher prevalence of cardiovascular disease and increased risk of all-cause mortality (Lechat et al., 2022; Gaffey et al., 2025; Hoog et al., 2026). COMISA patients also manifest a greater burden of depressive symptoms and psychological distress (Lang et al., 2017; Yang et al., 2010). Nevertheless, COMISA remains underrecognized. These patients may report substantial sleep disruption despite less pronounced upper airway collapsibility and may poorly tolerate CPAP (Sweetman et al., 2021a; Brooker et al., 2023), prompting consideration of adjunctive approaches, such as cognitive behavioral therapy for insomnia or hypnotics. Thus, subjective sleep quality metrics should be incorporated into a multidimensional evaluation of OSA (Figure 1).

#### 2.2.2 Daytime symptoms: sleepiness, fatigue, and quality of life

Among patients with similar AHI-defined disease severity, cluster-based analyses have consistently identified distinct symptom phenotypes, most commonly characterized as: (1) an “excessively sleepy” phenotype marked by high levels of daytime sleepiness and impaired alertness; (2) a “disturbed sleep” or “insomnia-like phenotype” characterized by frequent awakenings, poor sleep quality, and fatigue; and (3) a “minimally symptomatic” phenotype with limited perceived symptoms despite significant physiological disease burden (Keenan et al., 2018; Ye et al., 2014; Pien et al., 2018). These phenotypes differ not only in symptom expression, but also in demographic and clinical characteristics, including age, sex distribution, comorbidity burden, and behavioral profiles.

Most importantly, these symptom-based phenotypes also differ in clinical outcomes and treatment response. The excessively sleepy phenotype has been associated with a higher risk of adverse cardiovascular outcomes compared with other phenotypes (Mazzotti et al., 2019), suggesting that symptom manifestation may capture clinically relevant disease burden, which are not reflected by AHI alone on a single night. Additionally, treatment adherence varies by phenotype. Patients with excessive daytime sleepiness are more likely to adhere to CPAP therapy, whereas minimally symptomatic or COMISA patients demonstrate lower adherence and may derive less perceived benefit from treatment (Sweetman et al., 2021a; Zinchuk and Yaggi, 2019).

Beyond physiological measures, OSA has substantial psychosocial consequences that are often underappreciated in clinical practice. Patients frequently report impaired ability to function at work, reduced engagement in daily activities, and strain in interpersonal relationships, including difficulty sharing a bed due to snoring. These issues are often neglected in the clinical and research setting, despite being the chief complaint in patients seeking treatment for OSA (Mallampalli et al., 2026; Malhotra et al., 2020; Hoffstein et al., 1995). QoL impairments are often poorly correlated with AHI (Jackson et al., 2018), further emphasizing the limitations of relying on event frequency alone. Notably, sex differences are observed, in which women typically present lower AHI despite reporting greater impairments in QoL than men (Mallampalli et al., 2026), as well as higher rates of depression and anxiety (Wimms et al., 2024).

Taken together, these findings highlight that recognition of distinct symptom phenotypes and their underlying mechanisms supports a more individualized approach to OSA assessment and management, incorporating both physiological and patient-reported dimensions of disease burden. We propose that a more comprehensive subjective evaluation of symptoms (daytime sleepiness, fatigue, snoring, subjective sleep quality) and assessment of impairments in daytime functioning may also provide better association with objective measures of OSA burden, assisting in the OSA risk stratification and analysis of treatment responses (Figure 1).

## 3 Final considerations: a call to action in OSA field

Considering the marked phenotypic heterogeneity of OSA, reliance on AHI as a single metric is insufficient. In the present perspective, we discuss a new multidimensional evaluation of OSA patients to better capture inter-individual variability in pathophysiology, symptom burden, and risk of long-term sequelae, which includes the measurement of (but is not limited to) (Figure 1):

(1) Upper airway obstruction (e.g., AHI),
(2) CIH (e.g., HB)
(3) Nighttime symptom burden (e.g., snoring and subjective sleep quality)
(4) Daytime symptom burden (e.g., sleepiness and fatigue)

These metrics have not only shown strong predictive values for cardiovascular outcomes in OSA, but they also provide insights on the variability in treatment responses according to underlying OSA physiological burden. In addition, effectiveness of therapies, such as CPAP, is constrained by adherence, underscoring the importance of considering exposure-adjusted metrics, such as the mean disease alleviation (MDA), when evaluating OSA treatment success. MDA was developed as an objective measure of therapeutic effectiveness, calculated as the product of therapeutic efficacy (the percentage reduction in AHI) and objective adherence (the proportion of total sleep time on therapy) (Vanderveken et al., 2013). Reference values have recently been described, with a mean MDA of approximately 54% after 6 months of CPAP, and an MDA of at least 40% associated with clinically meaningful improvements in daytime sleepiness (Budhiraja et al., 2025). Notably, MDA is derived solely from AHI and adherence and does not directly incorporate patient-reported symptoms. In this sense, an extended three-dimensional MDA (3D-MDA) has been proposed to integrate symptoms domain, consistent with the multidimensional approach advocated here (Kaffenberger et al., 2025). In several other heterogeneous chronic diseases, such as chronic obstructive pulmonary disease (COPD), multiple sclerosis, and rheumatoid arthritis, the clinical assessment has evolved from reliance on a single metric toward integrated models that incorporate multiple domains of disease pathophysiology and patient outcomes (Fischer et al., 1999; Johnson et al., 2020; Corlateanu et al., 2021). For example, in COPD, the Global Initiative for Chronic Obstructive Lung Disease (GOLD) classification integrates symptom burden with objective measures, such as airflow limitation and exacerbation risk, enabling more accurate risk stratification and treatment personalization (Lange et al., 2012; Soriano et al., 2013). Methodologically, development of a multidimensional disease-severity index for a heterogeneous chronic disease would typically involve: (1) defining domains through literature review and expert/stakeholder consensus, (2) selecting and weighting candidate measures within each domain, and (Young et al., 2008) prospective testing of the composite for discrimination, reliability, construct validity, responsiveness to treatment, and prediction of clinically meaningful outcomes (Jones et al., 2016).

The multidimensional evaluation of OSA proposed here is conceptually aligned with other clinical frameworks, such as the Baveno classification (Randerath et al., 2021; Matthes et al., 2024), regarding the comprehensive combination of symptom burden and long-term sequalae risk. However, it may add complementary value to the current literature. Firstly, beyond symptoms and comorbidities, it emphasizes objective measures of hypoxic burden. Secondly, whereas Baveno is primarily oriented toward categorizing baseline disease burden in the context of other comorbidities and prioritizing patients for treatment, the present approach is also intended to characterize treatment response and clinical meaningfulness by tracking changes across these objective and subjective domains rather than relying on AHI normalization alone. Therefore, the multidimensional evaluation proposed in the present perspective (Figure 1) provides a comprehensive assessment of OSA by integrating both physiologic and symptomatic burden to define disease severity and treatment response with potential clinical application in improving risk stratification and optimizing treatment based on patient-centered outcomes. AHI, HB and symptoms should be considered complementary rather than interchangeable markers of disease burden. Discordant phenotypes may occur, including patients with low AHI but elevated HB, reflecting prolonged or deeper desaturation events despite fewer respiratory events, and patients with high AHI and symptom burden, but relatively low HB, reflecting frequent events associated with limited hypoxemia. Treatment prioritization should therefore integrate both indices alongside symptom burden and clinical context.

Looking ahead, a future framework grounded in multidimensional evaluation of OSA will require thorough validation and ongoing discussion before it can be widely adopted in clinical practice. Key questions remain regarding the integration of proposed metrics for grading OSA risk and guiding treatment responses, including the refinement of clinically meaningful thresholds for measures, such as the HB. Studies have identified HB above approximately 60%min/h as a candidate threshold associated with increased cardiovascular morbidity and mortality (Azarbarzin et al., 2019; Peker et al., 2025). However, reported cut-offs have varied across cohorts and a large European cohort study showed a dose-dependent cardiovascular risk increase without a clear inflection point or “safe” threshold (Trzepizur et al., 2022), underscoring the need for further validation and consensus. The relative contributions of objective and subjective dimensions are yet to be clarified, warranting additional exploration. Furthermore, while the use of metrics, such as HB has expanded within the OSA field, standardized definitions and analytical procedures across sleep centers have not been established, underscoring the necessity for consensus and uniformity. Any such multidimensional system will need to undergo prospective validation in large clinical studies to confirm its utility and relevance. Feasibility is an equally important consideration. Comprehensive symptom assessment, sleep studies, and phenotyping require additional time, expertise, and infrastructure that may be limited in resource-constrained healthcare systems.

Despite the remaining challenges, the current perspective provides a structured starting point to initiate a call to action in the OSA field, in which investigators, clinicians, and other health professionals are encouraged to rethink the way OSA severity and clinical management are faced. From a clinical perspective, meaningful treatment benefit should not solely rely on normalization of AHI, but rather combine AHI reductions with improvements in the physiological and symptomatic domains most relevant to the individual patient. This consideration is particularly important in patients with mild OSA, in which symptom burden, adherence, and long-term risk remain incompletely understood. As such, defining clinical meaningfulness in OSA requires moving beyond AHI to a comprehensive, multidimensional approach that reflects the totality of disease biology, patient experience, and treatment exposure. Future studies should develop and validate composite or personalized indices that align more closely with clinically meaningful outcomes across OSA phenotypes.

## Data Availability

The original contributions presented in the study are included in the article/supplementary material, further inquiries can be directed to the corresponding author.
